# Prevention and Immunotherapy of Secondary Murine Alveolar Echinococcosis Employing Recombinant EmP29 Antigen

**DOI:** 10.1371/journal.pntd.0003795

**Published:** 2015-06-08

**Authors:** Ghalia Boubaker, Andrew Hemphill, Cristina Olivia Huber, Markus Spiliotis, Hamouda Babba, Bruno Gottstein

**Affiliations:** 1 Institute of Parasitology, University of Bern, Bern, Switzerland; 2 Graduate School for Cellular and Biomedical Sciences, University of Bern, Bern, Switzerland; 3 Faculty of Pharmacy, Department of Clinical Biology B, Laboratory of Medical and Molecular Parasitology–Mycology (LR12ES08), University of Monastir, Monastir, Tunisia; The First Affiliated Hospital of Xinjiang Medical University, CHINA

## Abstract

Alveolar echinococcosis (AE) is caused by infection with the larval stage of the tapeworm *Echinococcus multilocularis*. An increasing understanding of immunological events that account for the metacestode survival in human and murine AE infection prompted us to undertake explorative experiments tackling the potential of novel preventive and/or immunotherapeutic measures. In this study, the immunoprotective and immunotherapeutic ability of recombinant EmP29 antigen (rEmP29) was assessed in mice that were intraperitoneally infected with *E*. *multilocularis* metacestodes. For vaccination, three intraperitoneal injections with 20μg rEmP29 emulsified in saponin adjuvants were applied over 6 weeks. 2 weeks after the last boost, mice were infected, and at 90 days post-infection, rEmP29-vaccinated mice exhibited a median parasite weight that was reduced by 75% and 59% when compared to NaCl- or saponin–treated control mice, respectively. For immunotherapeutical application, the rEmP29 (20μg) vaccine was administered to experimentally infected mice, starting at 1 month post-infection, three times with 2 weeks intervals. Mice undergoing rEmP29 immunotherapy exhibited a median parasite load that was reduced by 53% and 49% when compared to NaCl- and saponin–treated control mice, respectively. Upon analysis of spleen cells, both, vaccination and treatment with rEmP29, resulted in low ratios of Th2/Th1 (IL-4/IFN-γ) cytokine mRNA and low levels of mRNA coding for IL-10 and IL-2. These results suggest that reduction of the immunosuppressive environment takes place in vaccinated as well as immunotreated mice, and a shift towards a Th1 type of immune response may be responsible for the observed increased restriction of parasite growth. The present study provides the first evidence that active immunotherapy may present a sustainable route for the control of AE.

## Author Summary

Current medical management of AE that relies on surgery and continuous benzimidazole administration is of limited effectiveness. Therefore, alternative preventive and therapeutic tools need to be explored. Here, we demonstrate that vaccination with recombinant antigen EmP29 (rEmP29), prior or after secondary infection of BALB/c mice, resulted in a significant reduction of the median parasite weight when compared to different control groups. We then characterized the transcription level of splenic IL-4 and IFN-γ cytokines as hallmarks for AE-anti-protective humoral immune reaction (Th2) and for AE-effective (restrictive) cellular response (Th1), respectively. Results revealed that vaccinated mice in pre- or post-infection situation exhibited the lowest IL-4/IFN-γ mRNA ratios. In addition, those groups showed also significantly low levels of IL-10-encoding mRNA coding (immunosuppressive cytokine), as well as IL-2. These findings suggest that reduction of parasite load in rEmP29-vaccinated mice (in pre- or post-infection status) might be triggered by a decline of the immunosuppressive environment and a change of the host immune reaction towards a Th1-re-oriented cell-mediated immune defense. A similar non-specific effect appears also to be yielded by the immunostimulating adjuvants. This study provides the first insight into the potential benefits of antigen-specific immunotherapy as new treatment option of AE.

## Introduction

Alveolar echincoccosis (AE), caused by the metacestode larval stage of the fox tapeworm *Echinococcus multilocularis*, is one of the most severe helminthic diseases worldwide. Human AE affects the liver in more than 98% of the cases [1,2], and metacestodes grow and proliferate continuously and infiltratively, forming hepatic lesions that consist of parasite tissue, which is intermingled with host connective tissue and immune cells. Development of metacestodes affects liver homeostasis and causes irreversible granulomatous liver fibrosis [3–9]. Similar to malignant tumours, metastasis formation into other organs can take place at a later stage of infection [10].

Radical surgical removal of hepatic lesions is the optimal treatment option, but is feasible in only about 30% of the patients [11]. In advanced stages of AE, surgery is often incomplete due to the diffuse infiltration of metacestode tissue into non-resectable structures or sites [12].

The currently available chemotherapy is based on benzimidazole derivatives only, i.e. albendazole (ABZ) and mebendazole (MBZ) that target *E*. *multilocularis* β-tubulins [13]. However, despite that benzimidazole-based therapy has clearly increased the life expectancy of affected patients [12,14], its mode of action remains parasitostatic rather than parasitocidal [15]. *In vitro* studies have shown that ABZ has only restricted parasitocidial activity on *E*. *multilocularis* metacestodes [16,17]. Recently, it has been shown that stem cells of the *E*. *multilocularis* larval are expressing a particular β-tubulin isoform that is resistant to ABZ [18], which may partially explain the limited effectiveness of ABZ in the complete killing of the parasite [19,20].

From the immunological perspective, modulatory effects of ABZ on the host immune system has not yet been investigated. Thus, it may be that ABZ contributes to an increase of the anti-AE immune response [21]. For AE-patients who do not respond to or do not tolerate the benzimidazoles, no alternative drugs are available so far [22]. These limitations of the presently widely applied anti-AE chemotherapy have called for new or alternative or complementary or supportive therapy options, such as immunotherapy (e.g. complementary to chemotherapy).

Because of the high clinical and economic impact of human AE [23] on each patient, the development of protective and effective vaccines as a preventive measure against infection may represent an attractive alternative strategy. Vaccine candidates for inducing preventive protective immunity have been described so far, including e.g. recombinant Em 14-3-3 antigen that protects mice by 97% against *E*. *multilocularis* egg infection (primary AE) but not against challenge with *E*. *multilocularis* metacestodes (secondary AE) [24,25]. EG95 is an *E*. *granulosus* antigen that exhibited high-level protection against egg infection in sheep [26,27]. Immunization of mice with the homologous *E*. *multilocularis* EM95 antigen resulted in protection rates ranging between 78.5 and 82.9% [27] which was lower than that obtained using the EG95 antigen in sheep [26,28]. Recently, seven members of a tetraspanin transmembrane protein family (TSP1 to TSP7) have been shown to exhibit varying protective effects against primary AE. In infected BALB/c mice [29], the highest and lowest rates of lesion reduction were 87.5% and 37.6%, achieved by vaccination with TSP1 and TSP7, respectively [29,30].

The survival strategy of *E*. *multilocularis* is fundamentally based on its ability to induce anergy / immune tolerance in the host, by exerting potent and selective immunomodulatory activity [2]. The infection with *E*. *multilocularis* triggers an immune response that is characterized by an increasing imbalance between an initial rather cellular (Th1) and a subsequently prevailing humoral (Th2) immune response [2,3]. In the experimental mouse model, during the initiation of the infection, (early stage AE) both Th1 (IFN-γ) and Th2 (IL-4) related cytokines are present; but an initial dominance by Th1 cytokines and chemokines has been evidenced [5,6]. Subsequently (middle stage), an increase of mRNA levels of IL-4, IL-5, CCL8, CCL12, and CCL17 (Th2 cell-associated cytokines and related chemokines) is developing [6]. An increasing anergic immune status is evolving during late stage of infection, with an increase of Th2-oriented cytokine patterns, and an increase of immune-down regulating processes modulated by Tregs [6,7]. High transcription levels of IL-10 and TGF-β go in line with this anergic late-phase AE immune-status [3]. Indeed, the current evidence suggests that protection against *E*. *multilocularis* infection is basically associated with the maintenance of a Th1-oriented cellular immune response, while an increasingly dominating Th2 profile has been associated with a rather progressive form of AE in humans [31,32], although full metacestode proliferation capacity only occurs when cell-mediated immunity completely fails such as experienced with AIDS [33]. In a similar context, it has been recently shown that individuals under immunosuppressive therapy for cancers, autoimmune diseases or subsequent to liver-transplantation are at significant risk for *E*. *multilocularis* infection associated with a delayed diagnosis. In those patients, progression of AE occurs faster than in non- immunocompromised patients [34]. It has been suggested that *E*. *multilocularis* actively governs the immunological orientation of the host through up-regulation of immunosuppressive cytokines, mainly IL-10 [35] and TGF-β [7,36]. This may occur via metabolites, and immunomodulatory proteins such as EmTIP secreted by *E*. *multilocularis* during the very early stage of metacestode development [37] may accordingly be key protagonists. The laminated layer (LL) represents the most outer component of the *E*. *multilocularis* larvae, and LL-associated carbohydrate antigens such as Em2(G11) and Em492 [31,38,39] were shown to exhibit suppressive effects on concanavalin A—mediated proliferation of spleen cells from *E*. *multilocularis*-infected mice. Thus components of the LL could be involved in modulating the Th1/Th2 balance by driving it from a Th1-domination to a rather Th2-oriented control characterized by anergy promoted via Tregs and other immunomodulating parameters.

The working hypothesis for the present study was based on the work published by Harraga et al. [40] that a support of the host to maintain, or re-orientate, its immune response at a Th1-level may contribute to restrict or inhibit metacestode growth. Immunotherapy can either be active (or antigen-specific) or passive (non-specific). In non-specific immunotherapy, immune molecules are administrated to infected individuals to modulate an existing, but non-protective, immunity towards an adequate and effective response. Cytokines that promote differentiation of Th1 cells such as IL-12 [41] and IFN-α-2a [40,42] have been assessed for passive immunotherapy of AE in experimentally infected mice. Passive cytokine therapy based on rIFN-γ was also evaluated in human and murine AE [43–45]. Although Th1 cytokine-mediated therapies resulted in partial inhibition of the parasite growth in mice and in clinical stabilization of AE in human patients, such treatments do not generate immunologic memory and may lead to non-specific inflammation, which in turn can cause severe adverse effects.

An alternative approach, referred to as active immunotherapy, is to administer potential target antigens, and thus to elicit a specific immune response and immune memory. A high potential for such therapeutic vaccines directed against various pathogens has been reported, including rabies virus [46], *Mycoplasma pulmonis* [47], *Mycobacterium tuberculosis* [48], *Leishmania major* [49], and *Schistosoma mansoni* [50]. These studies demonstrated that administration of antigen doses (alone or in combination with additional chemotherapy in the case of rabies [46] and *Schistosoma* [50] could clear or greatly reduce an ongoing infection by the respective pathogen. It is now believed that therapeutic vaccines against chronic infectious diseases may overcome the potential impairment of immune responses due to an established infection [51]. However so far, no studies focused on creating and assessing therapeutic vaccines for the treatment of AE.

The P29 protein has been identified in the larval stage of both *E*. *granulosus* [52,53], and *E*. *multilocularis* [54]. Prior studies suggested a possible role of *E*. *granulosus* P29 (EgP29) as developmentally regulated component of the *E*. *granulosus* metacestode [55]. Vaccination of mice with bacterially produced recombinant EgP29 (rEgP29) was shown to lead to significant protective immunity, resulting in 96.6% protection against challenge infection with *E*. *granulosus* protoscoleces [56]. In this study, we examined the antigenicity and immunogenicity of recombinant *E*. *multilocularis* P29 (rEmP29), and applied rEmP29 either as a vaccine (prevention of infection) or as an active immunotherapy (treatment of infection).

## Materials and Methods

### Ethical statement

Female BALB/c mice, 8–10 weeks old were obtained from Charles River GmbH, Germany. All mice were housed und handled under standard aseptic animal laboratory conditions according to the Swiss Animal Welfare regulations (license No. Be108/08). For all experiments, animals were matched for age and weight. Experimental groups of 6 animals each were used.

### Maintenance of *E*. *multilocularis* metacestodes

The *E*. *multilocularis* isolate H95 [57] was used in this study. Metacestodes were maintained in BALB/C mice by serial transplantation passages as previously described [58]. For experimental infections, vesicle suspensions were prepared as described earlier [59], and mice were intraperitoneally (i.p.) injected with 100 μl vesicle suspension (corresponding to approximately 50 metacestodes).

### Recombinant antigens

The recombinant *E*. *multilocularis* P29 (rEmP29) protein was expressed and purified as previously described. [60]. Recombinant *Neospora caninum* microneme protein 1 (rNcMIC1), used here as an irrelevant control antigen expressed and purified under identical conditions, was produced as previously described [61]. Purified rEmP29 and rNcMIC1 were dialysed against PBS for two days at 4°C and kept at -80°C for subsequent use in serological assays. Prior to the use in cell cultures or mouse experiments, recombinant antigens were purified by affinity chromatography employing a Detoxi-gel affinity pack column (Pierce, Rockford, IL USA) to remove endotoxins. Protein concentration was determined by the Bio-Rad Bradford protein assay (Bio-Rad Laboratories GmbH, Germany). No measurable endotoxins were detected using Pierce LAL Chromogenic Endotoxin Quantitation test (Thermo Scientific, Rockford, IL, USA) conducted according to the manufacturer's protocol.

### Animal experimentation

Three experiments were performed. In Experiment 1, mice were vaccinated with rEmP29 and the immune response towards vaccination was investigated. In experiment 2, mice were vaccinated and then experimentally infected by intraperitoneal (i.p.) injection of *E*. *multilocularis* vesicles to evaluate the immunoprotective potential of rEmP29 (experiment 2). In Experiment 3, mice were infected by (i.p.) injection of *E*. *multilocularis* metacestode vesicles, and animals were treated at one month post-infection by (i.p.) injection of rEmP29 antigen in order to assess the immunotherapeutic potential of rEmP29.

For the present mouse experiments, saponin was chosen as adjuvant since it appears as the most widely used adjuvant in previous research studies on vaccination against *E*. *multilocularis* [24] or *E*. *granulosus* [27,62]. In addition, Quillaja saponins are potent adjuvants that enhance both cellular and humoral immune responses [63,64].

For experiment 1 (assessment of immunogenicity of rEmP29), 18 mice were divided into 3 groups. On days 1, 14, and 28, post-vaccination, all mice from group 1 (rEmP29-Sap) were i.p. injected with 20 μg rEmP29 plus 50 μg saponin adjuvant (Sigma-Aldrich, Buchs, Switzerland), formulated in sterile saline solution (0.09% NaCl) such as to yield a final volume of 100μl. Mice from group 2 were administered 50 μg saponin in saline solution (adjuvants control = Sap), and mice from group 3 received only sterile saline solution (infection control = NaCl). On day 42, all mice were euthanized, sera and spleen were collected and analysed for antibody and cytokine responses.

For experiment 2 (assessment of immunoprotective potential of rEmP29), 24 animals were divided into 4 groups. On days 0, 14, and 28 (Table 1), all mice from group 1 (rEmP29-Sap/Inf) were i.p. injected with 20 μg rEmP29 plus 50 μg saponin adjuvant, formulated in sterile saline solution (0.09% NaCl) in a final volume of 100μl. Mice from group 2 were administered 50 μg saponin in saline solution (Sap/Inf = adjuvants control), and mice from group 3 received only sterile saline solution (NaCl/Inf = infection control). In addition, another control group (group 4) was introduced, mice of which received 100 μl saline solution containing 20 μg rNcMIC1 plus 50 μg saponin (rNcMIC1-Sap/Inf control). Two weeks after the last immunization, all mice were challenged by i.p. injection of 100 μL *E*. *multilocularis* vesicle suspension. At 3 months p.i., all mice were euthanized, followed by careful removal of the metacestode tissue from the peritoneal cavity. The parasite mass was immediately determined on a Mettler AE160 scale (Mettler Toledo AG, Greifensee). Spleen and blood samples were also taken for further analyses. Spleens were taken and placed in Hanks' balanced salt solution on ice. Blood samples were allowed to clot at room temperature for 30 min and then were centrifuged for 10 minutes at 3000 rpm.

**Table 1:** Preventive immunization and immunotherapy with rEmP29 antigen against secondary AE in BALB/c mice.

	Immunization			Inf.	Treatment			Sacrifice/analysis
	D0	D14	D28	D42	D73	D87	D101	D132
**Preventive vaccination**								
Group 1 (rEmP29-Sap)	x	x	x	x	-	-	-	x
Group 2 (Sap)	x	x	x	x	-	-	-	x
Group 3 (NaCl)	x	x	x	x	-	-	-	x
Group 4 ((rNcMIC1-Sap)	x	x	x	x	-	-	-	x
**Therapeutic vaccination**								
Group 1 (rEmP29-Sap)	-	-	-	x	x	x	x	x
Group 2 (Sap)	-	-	-	x	x	x	x	x
Group 3 (Sap)	-	-	-	x	x	x	x	x
Group 4 ((rNcMIC1-Sap)	-	-	-	x	x	x	x	x

D: days, Inf.: infection, sap: saponin adjuvant.

For experiment 3 (assessment of immunotherapy with rEmP29), 24 mice were infected i.p. with 100 μl *E*. *multilocularis* vesicle suspension. At 1 month post infection (p.i.), mice from group 1 (Inf/rEmP29-Sap) were i.p. injected with 20 μg rEmP29 plus 50 μg saponin adjuvant, emulsified in sterile saline solution (0.09% NaCl) for a final volume of 100μl. Mice from group 2 were administered 50 μg saponin in saline solution (Inf/Sap = adjuvants control), mice from group 3 received only sterile saline solution (Inf/NaCl = infection control), and mice from group 4 were treated with 100 μl saline solution containing 20 μg rNcMIC1 plus 50 μg saponin (Inf/rNcMIC1-Sap-control = irrelevant antigen control). These treatments were repeated 14 and 28 days later (days 87and 101, p.i., Table 1). At 3 months p.i., mice were euthanized and parasite mass was assessed as in experiment 2. Spleen and blood samples were also collected for further analyses, as described below. Infections, euthanasia and sample collection time points were synchronized between mouse experiment 2 and 3 (Table 1).

An additional control group (non-infected) consisted of 6 female mice, animals were maintained in the same conditions till the end as outlined for experiments 2 and 3. This control group was also sacrificed and blood and spleens were taken for the same further analyses.

### Lymphocyte proliferation assay

For experiment 1 (see above), spleen cells were collected by mincing spleen tissue and passing it through sterile 40μm-mesh stainless steel sieves. Erythrocytes were depleted using red blood cell lysis solution (Miltenyi Biotec, Germany) for 10 minutes, and the residual spleen cells containing amongst others T-cells, dendritic cells, B-cells and macrophages, were then suspended in RPMI 1640 complete culture medium, (Gibco BRL, Basel, Switzerland) including 10% heat-inactivated fetal calf serum (FCS; Biochrom, Berlin, Germany), 0.05 mM 2-mercaptoethanol (Sigma-Aldrich, Buchs, Switzerland), 2 mM L-glutamine (GibcoBRL), and 100 U of penicillin plus 50 mg of streptomycin per ml (GibcoBRL). Cell suspensions were distributed in polystyrene 96-well flat bottom sterile plastic plates (Greiner Bio-One; HuberLab, Aesch, Switzerland) at 2×10^5^ cells/100μl/well, and spleen cells were stimulated with rEmP29 at 0.1μg/2μL/well (1 μg/mL). Wells containing cell suspensions that were left unstimulated or stimulated with Conavalin (Con A triggers T- lymphocyte proliferation [65] (Sigma-Aldrich, Buchs, Switzerland) with a concentration of 0.2 μg/μL/well 2 μg/mL were included as negative control and as an internal positive stimulation control, respectively. Experiments were performed in triplicates and cultures were maintained in a 37°C humidified chamber containing 5% CO_2_ for 72 hours. Spleen cells proliferation was assessed using the BrdU Cell Proliferation Assay kit (Calbiochem, Weidenmattweg, Switzerland) according to manufacturer's protocol.

In experiment 2 and 3 (see above), spleen cells were identically isolated, and single cell suspensions were either left unstimulated (maintenance in culture medium) or they were stimulated with Con A (2 μg/mL) and assessed using the BrdU Cell Proliferation Assay kit.

All experiments were performed in triplicates and the proliferative response was expressed as a stimulation index (SI) calculated using the equation: SI = median absorbance of cells stimulated with Con A or rEmP29/median absorbance of unstimulated cells.

### ELISA

Serum levels of rEmP29 antigen-specific IgG, IgG1, IgG2a antibodies induced by vaccination in experiment 1 were measured by direct rEmP29-ELISA. IgG1 and IgG2a antibody concentrations were used as markers of Th2 and Th1 immune responses, respectively. Serum IgG concentrations against rEmP29 and Em2(G11) in mice from experiments 2 and 3 were determined by ELISA as previously described [66]. Briefly, antigens were coated onto 96-well microplates at a concentration of 1μg/mL for rEmP29 and 0.57μg/mL of carbohydrate for Em2 (G11)-antigen [66]. Plates were incubated at 4°C for overnight. After three washes with washing buffer (1.5 mM KH_2_PO_4_, 10 mM Na_2_HPO_4_, 150 mM NaCl, 2.5 mM KCl, pH 7.4), the plates were blocked with blocking solution (washing buffer supplemented with 1% horse serum) for 30 min at 37°C and subsequently incubated with the mouse sera diluted at 1:100 in blocking buffer for 30 min at 37°C. AP-conjugated goat anti-mouse IgG, IgG1 or IgG2a (Sigma-Aldrich, Buchs, Switzerland) were used as the secondary antibody to detect bound antibodies. Finally, immune complexes were revealed by incubating with orthophenylene diamine (Sigma-Aldrich, Buchs, Switzerland) and 0.15% H_2_O_2_ for 30 min. The reaction was stopped by addition of 50 μL of 1 M NaOH to each well, and the absorbance at 405 nm was measured with a Microreader (model 550, Bio-Rad). All samples were run in triplicates.

### Quantitation of cytokine mRNA of IL-4, IL-2, IL-10 and IFN-γ by real-time RT-PCR

For experiments 1, 2 and 3, total RNA was prepared from approximately 30 mg of mouse spleen tissue, which was disrupted and homogenized using a FastPrep-24 homogenizer and lysic matrix tubes (both MP Biomedicals, Illkirch Cedex, France). RNA was isolated using the RNeasy mini kit with on-column DNA digestion (Qiagen, Basel. Switzerland), according to the manufacturer’s instructions. RNA purity and quantity were calculated using the Nanodrop 1000 spectrophotometer. Equal amounts of six RNA samples derived from mice of the same group were pooled. The cDNA synthesis was performed with 1μg total RNA using M-MLV Reverse Transcriptase RNase H Minus (Promega, Zurich, Switzerland) according to the manufacturer’s instructions.

Comparative quantification of IL-4, IL-2, IL-10, and IFN-γ mRNA in murine spleens was performed by real time PCR using *β-actin* as housekeeping gene for the qRT-PCR. Primers were purchased from Sigma-Genosys and are all described in Table 2. Real-time PCRs were performed using FastStart Essential DNA Green Master Kit (Roche, Rotkreuz, Switzerland), and 2 μL of diluted cDNA in the presence of 0.2 μM of each specific primer. Quantitative PCR was performed using the Rotor-Gene 6000 (Corbett Life Science). Samples (including cytokines and β-actin) were run in triplicates. The programme included a hold at 95°C for 15 min, 50 cycles each of denaturation at 95°C for 15 s, annealing at 55°C for 30 s, extension at 72°C for 30 s. Melting curves were generated by heating the samples from 50°C to 90°C.

**Table 2:** Primers used for quantitative PCR.

GenBank Target gene	Accession Number	Primer sequence (5‘-3‘)	Product length (bp)	Ref *
β-actin	NM_007393	F:AACTCCATCATGAAGTGTGA	248	[36,90]
		R:ACTCCTGCTTGCTGATCCAC		
IFN-γ	K00083.1	F:ACTCAAGTGGCATAGATGTGGAAG	167	[36,90]
		R: GACGCTTATGTTGTTGCTGATGG		
IL-10	NM_010548.2	F:GGTTGCCAAGCCTTATCGGA	191	[36,90]
		R:ACCTGCTCCACTGCCTTGCT		
IL-2	BC116845.1	F:CCTGAGCAGGATGGAGAATTACA	141	[36,90]
		R:TCCAGAACATGCCGCAGAG		

*Citations between brackets indicate the source of primers sequences.

### Statistical methods

Normality of distribution was checked with Shapiro-Wilk test in the software R version 3.0.1 (R core team, A Language and Environment for Statistical Computing, R Foundation for Statistical Computing, Vienna, 2013). One-way analysis of variance (ANOVA) was applied for comparisons of the parasite load among mouse groups followed by Bonferroni-adjusted P values calculated by Pairwise T-Test in R. A P-value of <0.05 was considered significant. Data was visualized by boxplot in Microsoft Office Excel 2010.

For the spleen cell proliferation assay, ELISA and cytokines expression, data were expressed as median ± standard error (S.E.) and examined for statistical significance with the Student's t-test. P-values of less than 0.05 were considered to be statistically significant.

## Results

### Immunological hallmarks of vaccination with rEmP29 in mice

**Fig 1:**
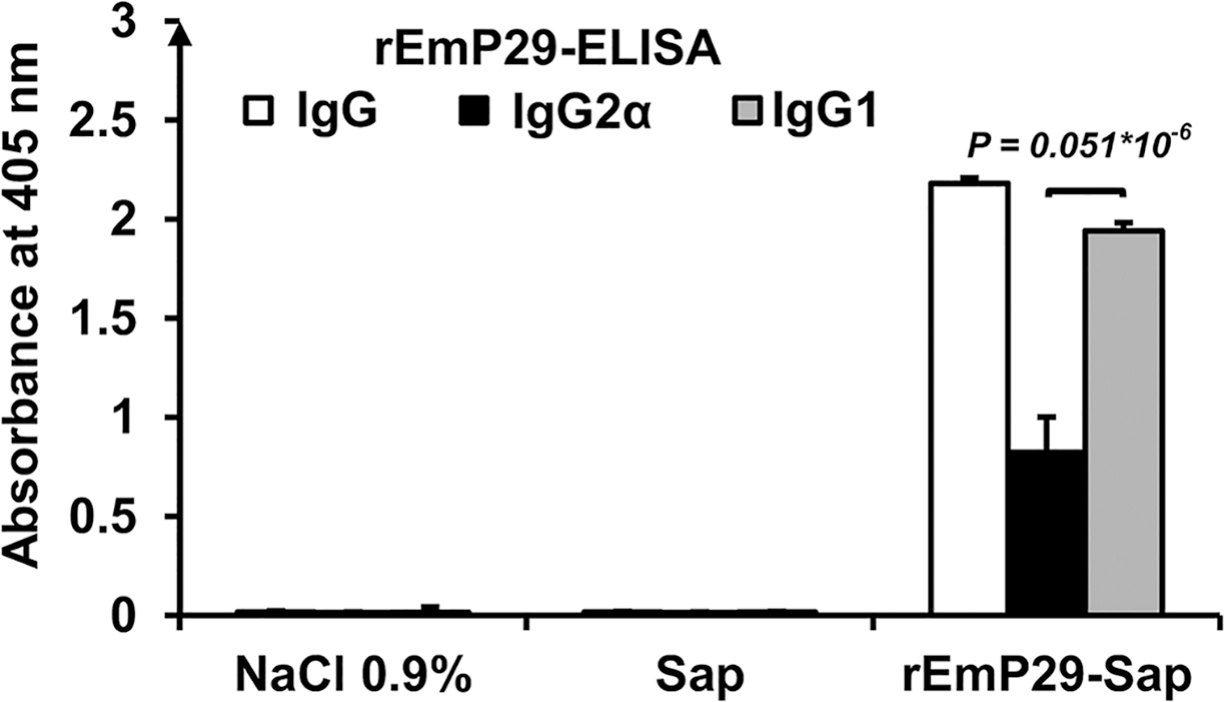
IgG isotype antibody responses in mice vaccinated i.p. with rEmP29 and saponin adjuvant. Antigen-specific ELISA was performed with sera collected 6 weeks after the first immunization. IgG, IgG1 and IgG2α results refer to median A404nm values + standard error (SE).

To determine the antibody responses and the subclass distribution of serum IgG antibody in mice immunized with rEmP29, serum levels of IgG, IgG2a and IgG1 were analysed. As shown in Fig 1, intraperitoneal immunization of mice with rEmP29 in combination with saponin resulted in a respectively specific anti-rEmP29 IgG antibody production. Furthermore, both tested IgG subclasses (IgG1 and IgG2α) were induced in rEmP29-Sap-immunized mice, with the IgG2a median OD value being significantly higher than that of IgG1 (P<0.001).

**Fig 2:**
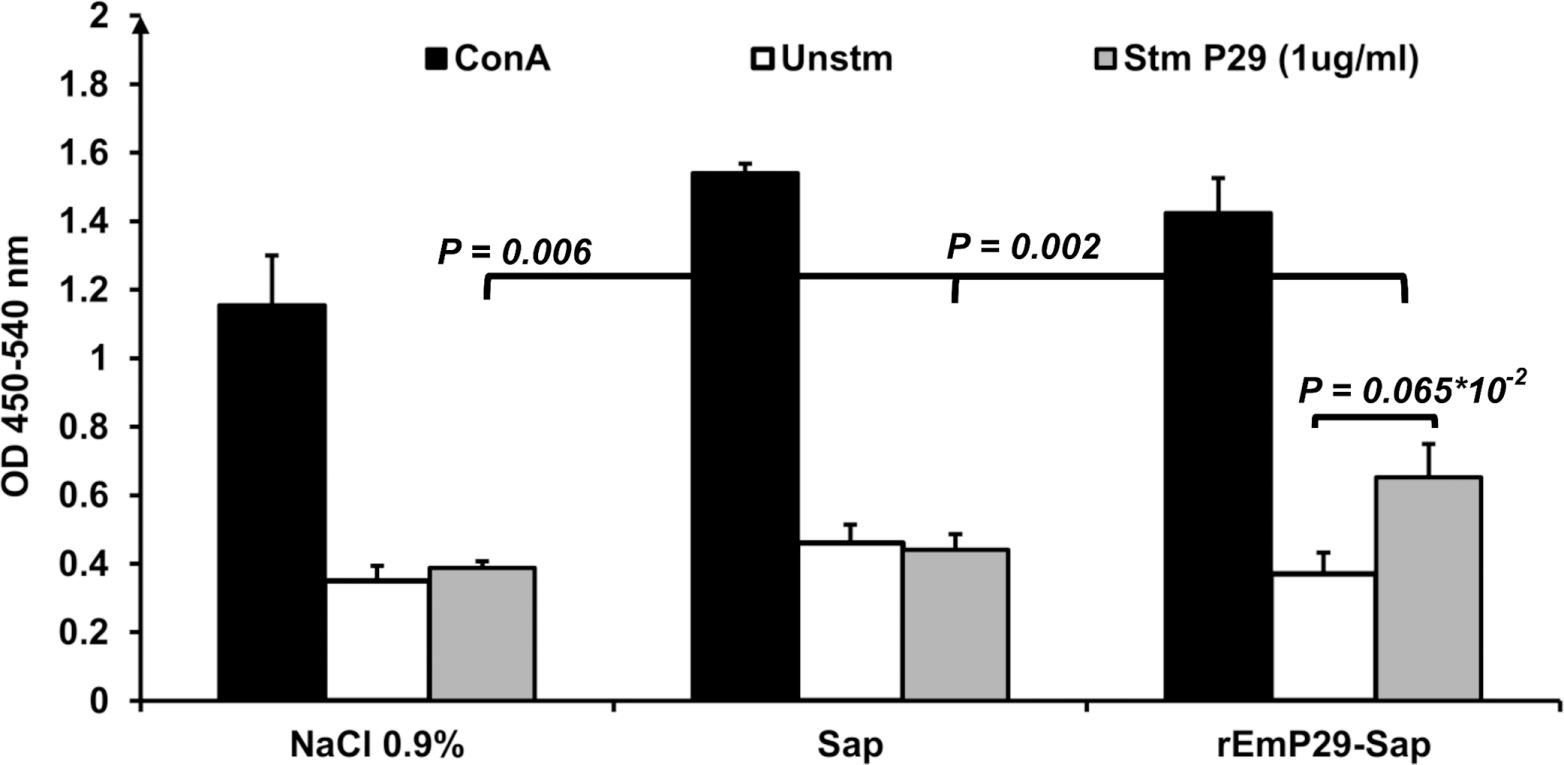
Measurement of spleen cell proliferation after mitogenic or antigen-specific stimulation of mice immunized with rEmP29 antigen. Two weeks after the final immunization, pooled spleen cells from the different immunized groups were cultured and stimulated separately with rEmP29 antigen (1 μg/mL) or with ConA (2 μg/mL), and proliferation levels were subsequently measured.

To examine the cellular immune response elicited by the rEmP29 vaccination, spleen cells proliferation was studied *in vitro* (Fig 2). In response to the rEmP29 specific antigen stimulation, spleen cells from rEmP29-vaccinated mice exhibited a significantly higher proliferation rate when compared to spleen cells from mice treated with saponin (*P = 0*.*002*) or saline solution (*P = 0*.*006*). Spleen cells from all animals responded to the polyclonal stimulant Con A as a positive proliferation control (Fig 2). Thus, priming with the rEmP29 vaccine led to specific proliferation of spleen cells, suggesting subsequent expansion of antigen-specific T cells.

**Fig 3:**
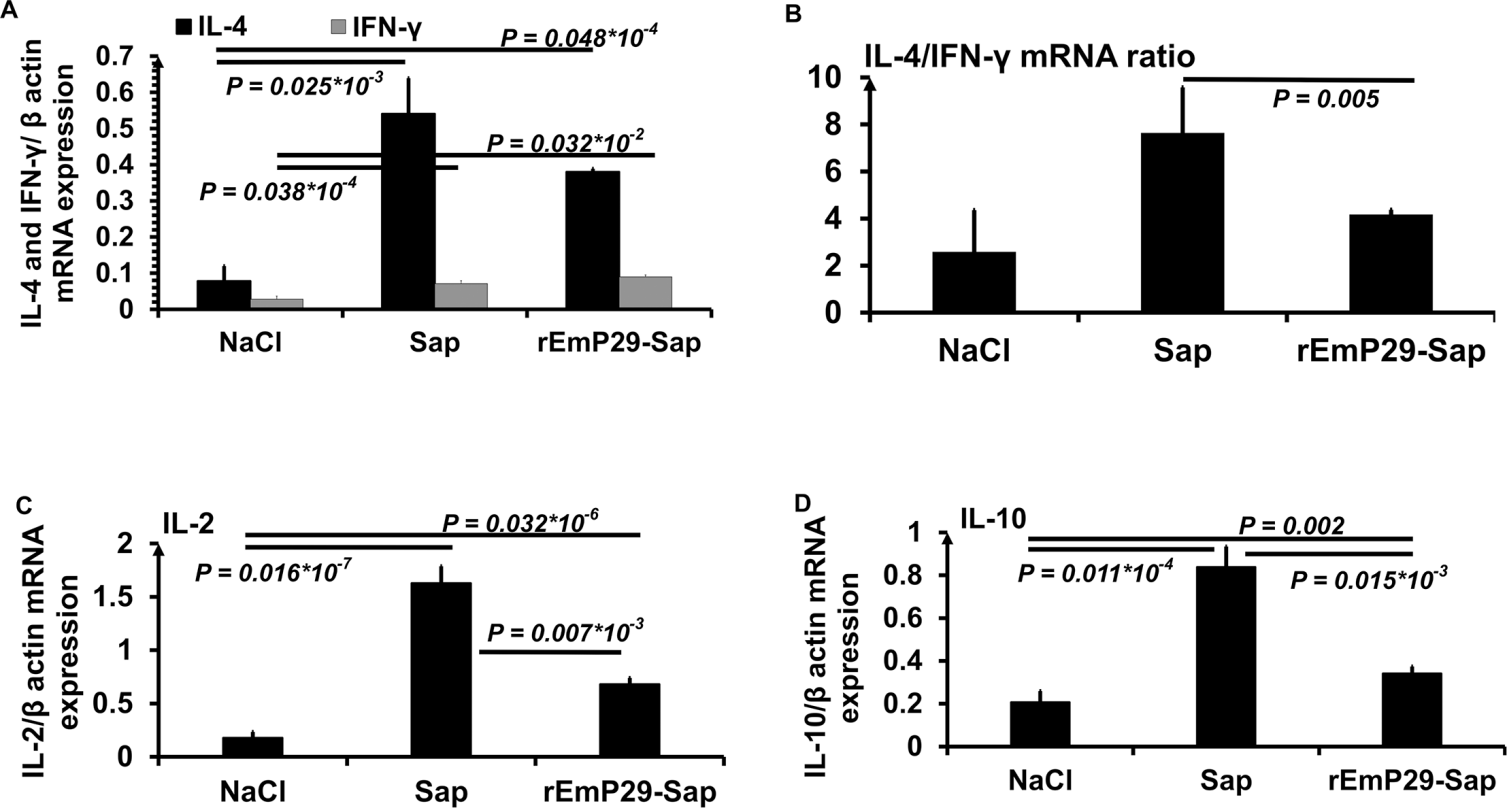
Relative mRNA expression levels of IL-4 (A), IFN-γ (A), IL-2 (C) and IL-10 (D) in spleens from BALB/C mice, following immunization with rEmP29 (rEmP29-sap) or with adjuvant alone (sap). The IL-4/IFN-γ cytokine mRNA ratios were calculated from the three groups **(B)** and indicate a Th2-dominated response in rEmP29-sap and sap-alone-treated mice compared to none immunized mice.

To characterize the cytokine expression profile in response to recombinant rEmP29 vaccination, we examined the mRNA levels of IFN-γ, IL-2, IL-4 and IL-10 in spleen cells by real-time RT-PCR. As shown in Fig 3A and 3B, immunization of mice with rEmP29 or with adjuvants alone (Sap) resulted in a significantly higher IL-4 (*P < 0*.*001)* and IFN-γ (*P < 0*.*001)* mRNA expression level when compared to spleens from saline-treated mice (NaCl) (Fig 3A and 3B).

IL-2 mRNA expression in spleen cells from mice immunized with rEmP29-Sap or saponin alone was significantly higher *(P < 0*.*001)* than that one observed in the control non-immunized animals (NaCl group) (Fig 3C). Similarly, IL-10 mRNA-level was significantly higher in rEmP29-Sap- and Sap-vaccinated mice when compared to the mouse group receiving only saline solution (NaCl group). However, the magnitude of IL-10 and IL-2 mRNA levels rising in rEmP29-Sap-immunized mice was lower than in mice inoculated with adjuvants alone (Fig 3D).

### Vaccination with rEmP29 antigen results in reduced parasite burdens in mice experimentally challenged with *E*. *multilocularis* metacestodes

**Fig 4:**
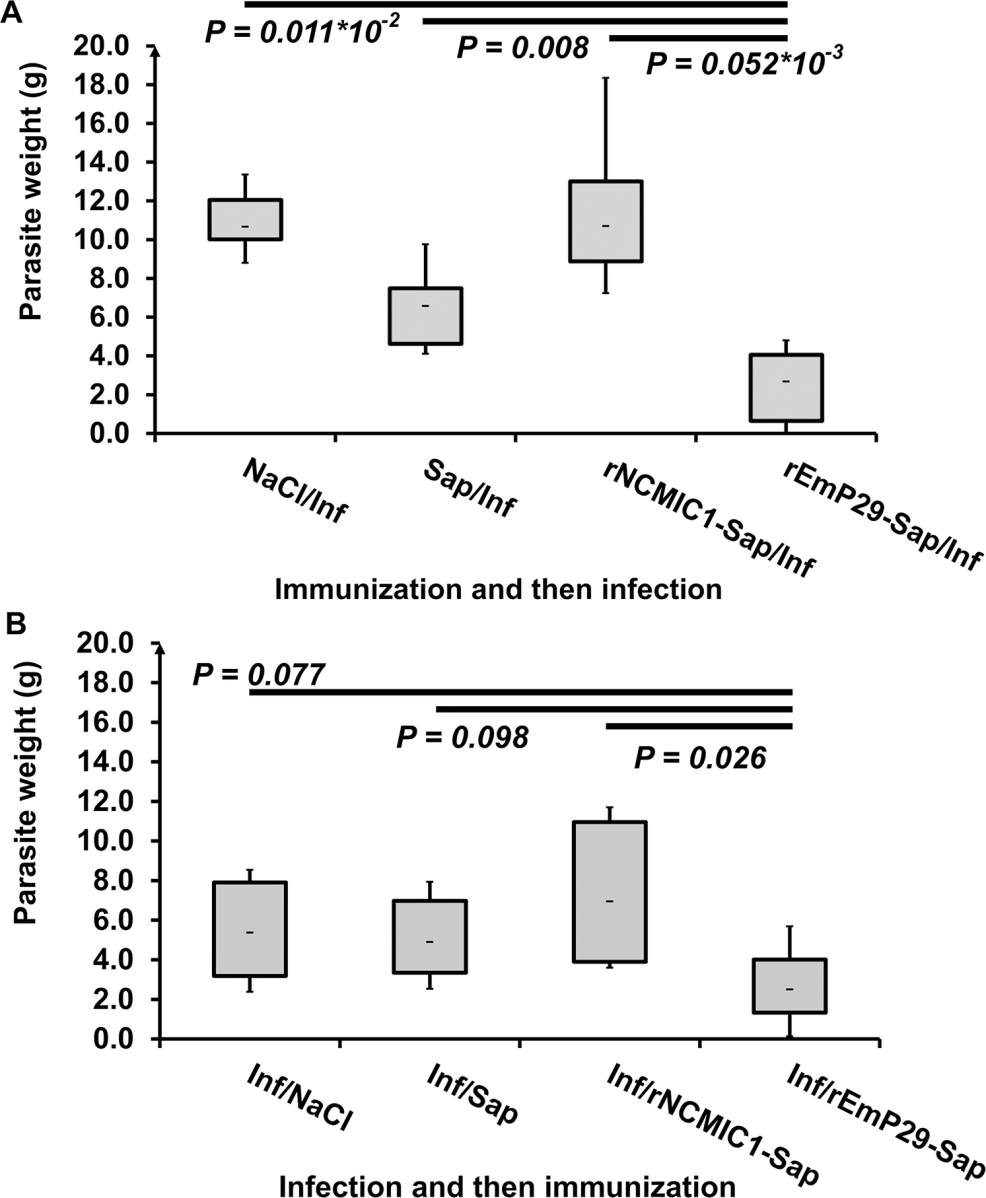
Therapeutic and protective effect of rEmP29 against secondary AE infection in BALB/c mice. **(A)** Assessment of parasite load in the intraperitoneal cavity of mice vaccinated i.p. with rEmP29-saponin, rNcMIC1-saponin or adjuvant alone and subsequently infected with *E*. *multilocularis* vesicle suspension. Mice were vaccinated three times (2 weeks apart), and at 3 months p.i. mice were euthanized and the total parasite mass recovered and weighed. **(B)** Assessment of parasite load in post-infection treated mice with rEmP29-saponin, rNcMIC1-saponin or adjuvant alone. Treatment started at 1 month after initial secondary infection. Shapiro-Wilk test suggested that the measured cyst weights followed normal distribution in all mouse groups. One-way ANOVA analysis proved a significant difference between the animal groups from experiment no. 2 (immunization before infection (A). Post-Hoc analysis by Pairwise T-Test with Bonferroni-adjustment showed that there was a significant reduction of the median parasite weight in rEmP29-Sap immunized group (A). For experiment no. 3 (B), significance was found only between rEmP29-Sap- and rNcMIC1-Sap-treated groups.

Recombinant EmP29 antigen was evaluated as a vaccine against *E*. *multilocularis* in the murine model of secondary AE (Fig 4A, Immunization and then infection). The efficacy was assessed by monitoring the parasite burden in the peritoneal cavity. In non-vaccinated saline treated mice, experimental infection resulted in a median parasite weight of 10.66 ± 1.68 g, while saponin treatment (6.57 ± 2.18 g) and vaccination with rNcMIC1 (10.70 ± 4.03 g) had no significant impact. In contrast, vaccination of mice by rEmP29 resulted in a significantly reduced median parasite bio-mass (2.68 ± 2.08 g), which was 75%, 59% and 75% lower when compared to NcMIC1-vaccinated, saponin-treated, or saline treated groups, respectively (Fig 4A).

### Immunotherapy with rEmP29 antigen in mice experimentally infected with *E*. *multilocularis* metacestodes results in reduced parasite burdens

To determine whether the course of an already established secondary *E*. *multilocularis* infection in mice could be altered by immune stimulation with rEmP29 antigen, three doses of rEmP29 formulated in saponin adjuvants were administered during the early phase of (after 1 month) (Fig 4B, Infection and then immunization). Mice that were infected and received only the saline solution developed a high median parasite load (5.37 ± 2.72 g). Similarly, mice that were treated with the adjuvant alone also developed high infection intensities (4.9± 2.31 g). Mice treated with rNcMIC1 antigen emulsified in saponin showed the highest median parasite burden (6.95 ± 3.81 g). In contrast, mice that were treated with three injections of rEmP29-saponin formulation on days 73, 87 and 101 (Table 1) post-infection exhibited a significantly lower parasite load (2.51± 2.07 g). In two mice the parasite loads (0.13 and 1.08 g) were largely inferior to the median. Thus immunotherapy with rEmP29 resulted in a reduction of the median metacestode weight of 53%, 49% and 64%, as compared to the NaCl-, saponin- and rNcMIC1-saponin-treated control mice, respectively. These differences were significant only upon comparison of rEmP29 and rNcMIC1 treated groups.

### Analysis of *E*. *multilocularis*-specific IgG antibodies responses to vaccination and immunotherapy in infected mice

**Fig 5:**
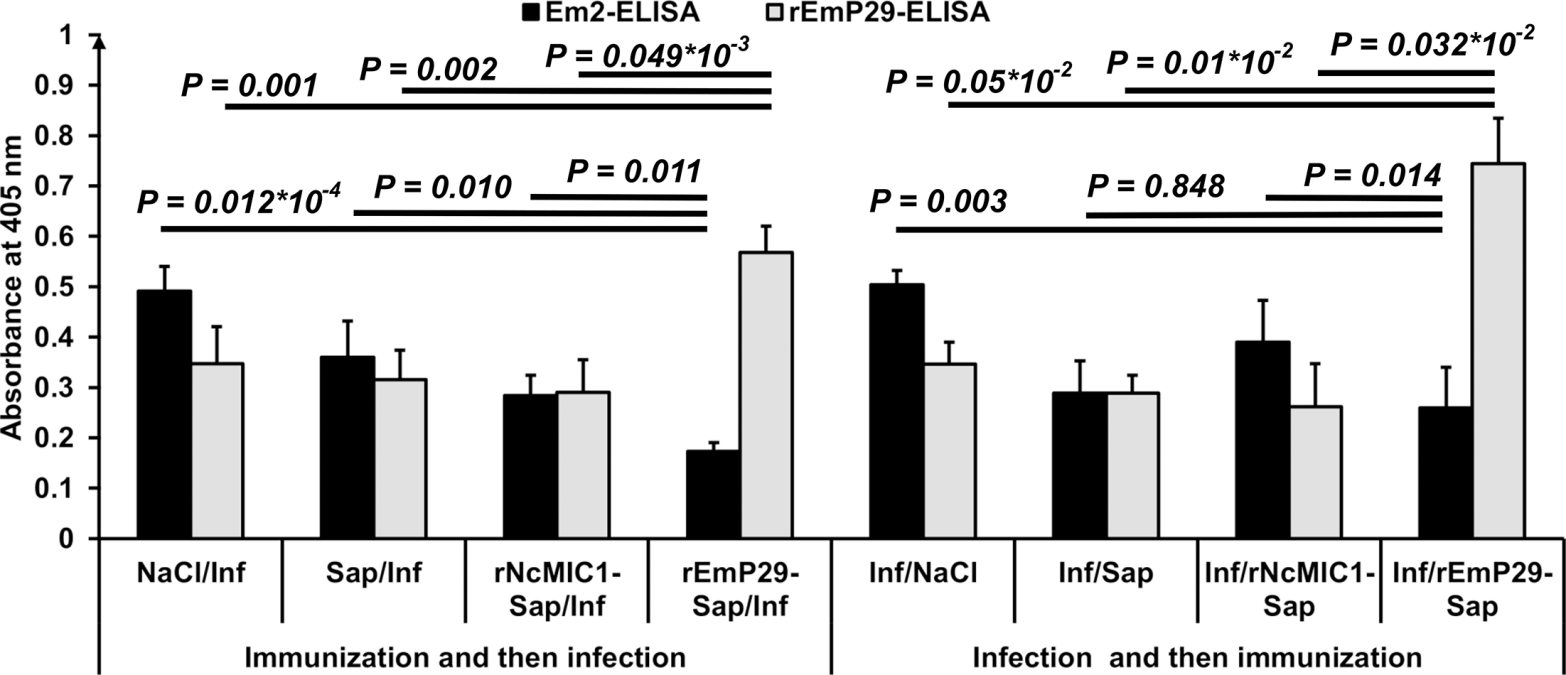
Serological analyses. Antibody responses of i.p. infected mice, vaccinated pre- or post-parasite challenge, were measured by ELISA using affinity-purified Em2 (G11) and rEmP29 antigen. Bars indicate SE. Immunization and then infection: mouse groups from experiment no. 2 were immunized with different preparations (Saline solution (NaCl), saponin (Sap), rEmP29 plus saponin (rEmP29-Sap) or with rNcMIC1 plus saponin (rNcMIC1-Sap)), and then challenged by infection with an *E*. *multilocularis* metacestode suspension. Infection and then immunization: mice from experiment no. 3 were infected with *E*. *multilocularis* metacestode suspension, and at 1 month p.i., animals were treated with different preparations (physiological saline solution (NaCl), saponin (Sap), rEmP29 plus saponin (rEmP29-Sap) or with rNcMIC1 plus saponin (rNcMIC1-Sap)).

In order to characterize humoral immune responses, sera of the different experimental groups in experiments 2 and 3were analysed by rEmP29 ELISA. All samples from *E*. *multilocularis* infected mice contained antibodies directed against rEmP29 protein, and the significantly highest anti-rEmP29 IgG-levels were detected in those animals that had been vaccinated with rEmP29-antigen and in mice that were treated with rEmP29 after being infected (Fig 5).

As shown in Fig 5, serum levels of the specific IgG response against the Em2 (G11) antigen was significantly lower in rEmP29 vaccinated mice, when compared to the corresponding groups immunized with saline solution only, saponin alone or with rNcMIC1-saponin.

Similarly, anti-Em2(G11)-specific IgG antibody levels in infected mice that were treated with rEmP29 were inferior to those in infected and treated animals with rNcMIC1 or left untreated. However, animals treated with saponin alone showed similar levels of anti-Em2 (G11)-specific IgG as compared to rEmP29-sap treated mice.

### Effects of EmP29-vaccination and immunotherapy on spleen cell proliferation and cytokine expression in experimentally infected mice

**Fig 6:**
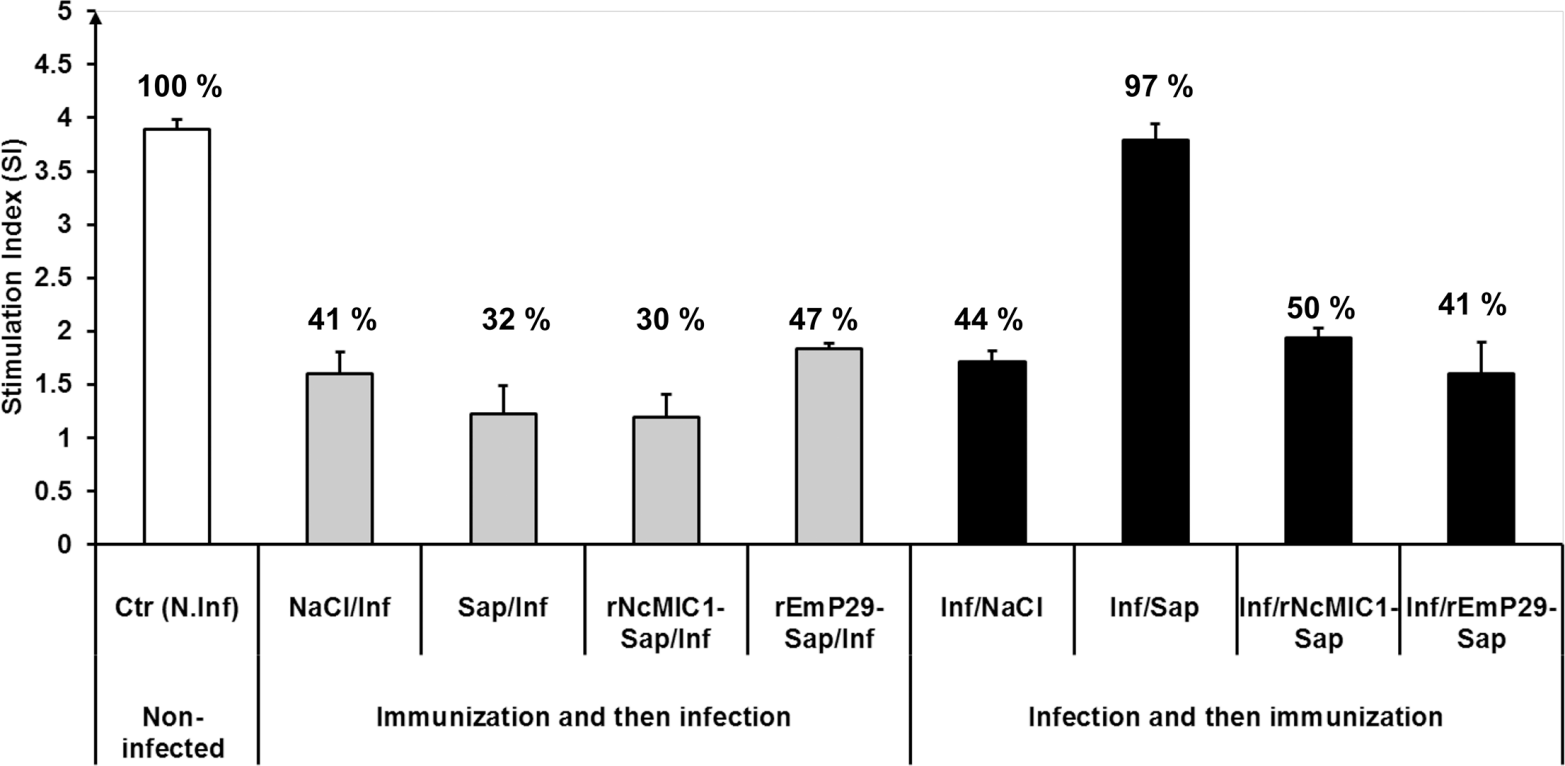
The proliferative responses to ConA stimulation of pooled spleen cells from non-infected control mice and from AE-infected mice (vaccinated with rEmP29 antigen and subsequently infected, or immunized with rEmP29 antigen at 1 month p.i.) were determined at 3 months p.i.. Stimulation indices (SI) were calculated as median ratio of A404nm values of the stimulated cells divided by values of untreated cells. The SI value obtained from non-infected mice was taken as 100% and SIs of other experimental groups were calculated in percentage to non-infected mice.(**), a statistically significant difference was found between non-infected mice and all remaining groups, except Inf/Sap. Immunization and then infection: mouse groups from experiment no. 2 were immunized with different preparations (saline solution (NaCl), saponin (Sap), rEmP29 plus saponin (rEmP29-Sap) or with rNcMIC1 plus saponin (rNcMIC1-Sap)), and then challenged by infection with *E*. *multilocularis* metacestode suspension. Infection and then immunization: mice from experiment no. 3 were infected with *E*. *multilocularis* metacestode suspension, and at 1 month p.i., animals were treated with different preparations (saline solution (NaCl), saponin (Sap), rEmP29 plus saponin (rEmP29-Sap) or with rNcMIC1 plus saponin (rNcMIC1-Sap)).

The proliferative response of spleen cells to ConA-mitogen is an important overall indicator of immune fitness. The proliferative responses of spleen cells to ConA stimulation from all groups (experiments 2 and 3 and including non-infected mouse group) are presented in Fig 6, by using the value of 100% as a reference proliferation of spleen cells (derived from non-infected mice). The proliferation indices of spleen cells from the four infected mouse groups of experiment no. 2; NaCl/Inf, Sap/Inf, rNcMIC1-Sap/Inf and rEmP29-Sap/Inf were 41%, 32%, 30% and 47%, respectively and were thus considerably (*P < 0*.*001*) lower than those observed in non-infected control groups (100%) (Fig 6).

In mouse experiment no. 3, spleen cell proliferation rates triggered by ConA were significantly (*P < 0*.*001*) reduced (44%, 50% and 41% in Inf/NaCl, Inf/rNcMIC1-Sap and Inf/rEmP29-Sap mouse groups, respectively). However, the proliferation of spleen cells in infected animals that were treated with saponin alone was similar (97%) to the non-infected control group (100%) (Fig 6).

**Fig 7:**
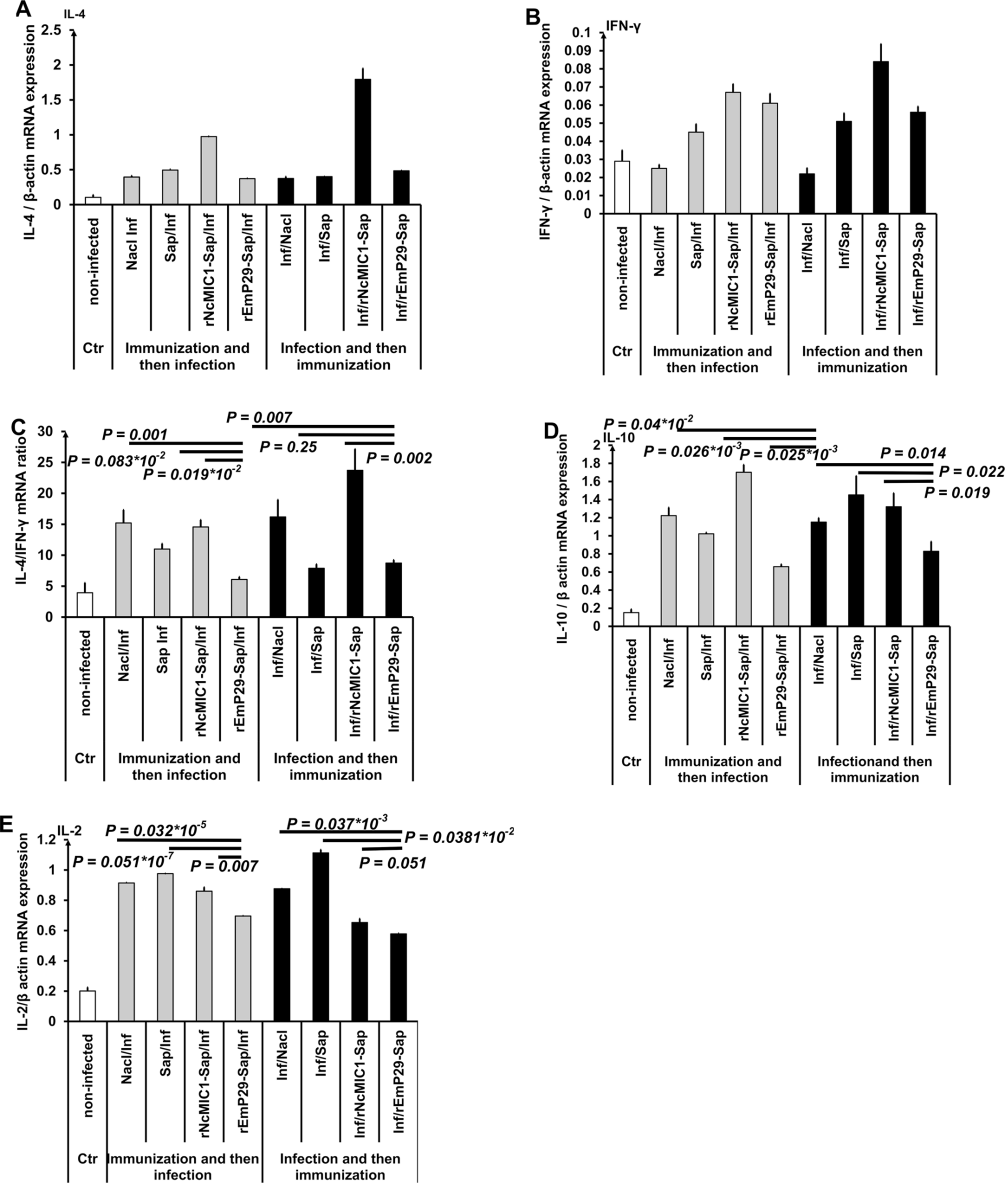
Cytokine gene expression levels of murine spleen cells following vaccination with rEmP29 in pre- (Immunization and then infection) or post-infection (Infection and then immunization) (A) Interleukin (IL)-4, (B) interferon (IFN)- γ, (C) IL-4/IFN-γ mARN ratios (D) IL-10 and (E) IL-2. Bars represent SE. Immunization and then infection: mouse groups from experiment no. 2 were immunized with different preparations (saline solution (NaCl), saponin (Sap), rEmP29 plus saponin (rEmP29-Sap) or with rNcMIC1 plus saponin (rNcMIC1-Sap)), and then challenged by infection with *E*. *multilocularis* metacestode suspension. Infection and then immunization: mice from experiment no. 3 were infected with *E*. *multilocularis* metacestode suspension and, at 1 month p.i., animals were treated with different preparations (saline solution (NaCl), saponin (Sap), rEmP29 plus saponin (rEmP29-Sap) or with rNcMIC1 plus saponin (rNcMIC1-Sap)).

The cytokine expression profiles in response to recombinant rEmP29 vaccination were investigated by comparing IFN-γ, IL-2, IL-4 and IL-10 mRNA levels in the spleen of the different treatment groups with cytokine mRNA levels in non-infected control mice by real time RT-PCR (Fig 7).

Significantly higher levels of IL-4 mRNA expression were detected in all 8 groups from experiments nos. 2 and 3, when compared to non-infected mice (Fig 7A).

In terms of IFN-γ expression, in rEmP29- vaccinated or-treated mice, IFN-γ mRNA levels were significantly elevated when compared to the corresponding groups treated with saline only (NaCl/Inf and Inf/NaCl groups) (Fig 7B). In addition, saline-treated mice in infected animals exhibited similar levels of splenic IFN-γ mRNA expression as non-infected mice (Fig 7B).

Furthermore, the IL-4 to IFN-γ mRNA expression ratio was calculated for each animal group and displayed as a graph (Fig 7C). Transcription of IL-4 mRNA in spleen cells from secondary AE-infected mice (NaCl/Inf and Inf/NaCl) was 16 times higher than that of IFN-γ (Fig 7C). In infected BALB/c mice that were vaccinated (rEmP29-Sap/Inf) or treated (Inf/rEmP29-Sap) with rEmP29, the ratio of IL-4/IFN-γ mRNA was lower than those of different control groups from experiments no. 2 and 3 (NaCl/Inf, Sap/Inf, rNcMIC1-Sap/Inf, Inf/NaCl, Inf/rNcMIC1-Sap). Nevertheless, treatment of infected mice with saponin (Inf/Sap) alone resulted in a similar IL-4/IFN-γ mRNA-ratio as found in animals that received rEmP29-Sap at 1 month post-infection.

In experiment no. 2, as shown in Fig 7D, infected mice (NaCl/Inf and Inf/NaCl) had significantly higher IL-10 mRNA expression levels than controls (non-infected) (*P < 0*.*001)*. In addition (Fig 7D), IL-10 mRNA transcription levels were significantly lower in rEmP29 vaccinated and infected mice (rEmP29-Sap/Inf), when compared to the following groups: NaCl/Inf, Sap/Inf and rNcMIC1-Sap/Inf. Similarly, immunotherapy treatment (experiment no. 3) of infected mice with rEmP29 in conjunction with the saponin adjuvant resulted in significantly reduced IL-10 transcription levels when compared to different control groups: Inf/NaCl, Inf/Sap and Inf/rNcMIC1-Sap (Fig 7D). The highest transcription level of IL-10 mRNA was observed in spleen cells from rNcMIC1-sap vaccinated mice (rNcMIC1-Sap/Inf group).

All infected groups (experiment nos. 2 and 3) presented significantly higher expression levels of IL-2 mRNA when compared to controls (non-infected) (Fig 7E). Infected mice that were immunized (experiment no. 2) or treated (experiment no. 3) with rEmP29 displayed a significantly lower amount of mRNA coding for IL-2, correspondingly compared to the following groups: only infected mice (NaCl/Inf and Inf/NaCl), infected mice that were either immunized or treated with adjuvant (Sap/Inf and Inf/Sap) or immunized with rNcMIC1-saponin (rNcMIC1-Sap/Inf group) as shown in Fig 7E.

## Discussion

This is the first study describing an antigen-based immunotherapy for experimentally induced secondary murine AE, based on the bacterially produced recombinant rEmP29 antigen. We also present data that demonstrates an explorative experiment tackling the efficacy of rEmP29 antigen as a vaccine candidate against experimental (secondary) *E*. *multilocularis* infection in mice. In addition, spleen cells recall-responses upon antigen stimulation and real time PCR-based cytokine mRNA expression analyses suggested that both, vaccination and immunotherapy, overrode to some extent the *Echinococcus*-mediated immunomodulation at the host-parasite interface. EmP29 was selected as a target antigen for two reasons: (i) EmP29 exhibits high expression levels in both *E*. *multilocularis* [54] and *E*. *granulosus* [52,53] metacestodes, indeed it was previously suggested that P29 could have an important role in developmental regulation of the *E*. *granulosus* metacestode [55]; (ii) recombinantly expressed *E*. *granulosus* P29 (rEgP29) vaccination provides effective protection of mice against challenge infection with *E*. *granulosus* protoscoleces [56].

Three injections of rEmP29-antigen formulated in saponin adjuvants prior to experimental infection lead to a significantly reduced (75%) parasite mass formation as compared to non-vaccinated control mice. In addition, intraperitoneal injection of rEmP29 antigen during the chronic phase of AE resulted in a partial control of parasite growth, and yielded a reduced the median parasite mass by 53% as compared to non-treated mice.

In contrast to the result obtained in our study, with rEmP29 applied as preventive vaccine against secondary AE, immunization of mice with the recombinant Em14-3-3 (E14t) in combination with a saponin adjuvans, did not show any reduction in the parasite load when compared to non-vaccinated animals [24]. This discrepancy between rEmP29 and E14t cannot yet be explained, but hypothetically the effect may be related to distinct biological function or by different antigenic and immunogenic properties of the reagents.

So far, the core biological function of EmP29 in the *Echinococcus* biology is still unresolved. An NCBI BLAST search showed that the amino acid sequence of EmP29 (GenBank, accession no. AAD53328.1) exhibited 100% homology to *E*. *granulosus* endophilin B (GenBank, accession no. CDJ21798.1) [18]. However, EmP29 shares only a low degree of identity (22%) with human endophilin B1 (S1 Fig). In eukaryotes, endophilin B1 is essential for synaptic transmission [67], and inhibition of its expression has been shown to produce profound defects in synaptic vesicle endocytosis [68]. However, the mechanism by which endophilin B promotes endocytosis has remained controversial. Therefore, the future functional characterization of EmP29 will be of great interest to understand the cellular pathways and functional activities associated with EmP29.

To induce experimentally murine secondary AE, *E*. *multilocularis* metacestodes are injected intraperitoneally into susceptible mice. Thus at the starting point of an experimental immunotherapy (one month p.i.), it is conceivable that metacestodes are already well established within the host, including a solid protection by the parasite-derived laminated layer [69,70]. In our experiments, the third and final injection of rEmP29 antigen was administered at 1 month before the end of the experiment. Thus, we do not know yet how the parasite growth-potential develops at later time points. Therefore, further animal studies are planned, during which different experimental starting- and end-points will be assessed. We hypothesize that the effectiveness of immunotherapy is largely dependent on the time point of the initiation of treatment in relation to the infection stage. For example in pythiosis in horses, caused by *Pythium insidiosum*, a successful immunotherapy depends on the chronicity of the lesions prior to immunotreatment. Therefore, all horses with lesions less than 15 days old were cured by an early *P*. *insidiosum*-antigen, while those with chronic pythiosis (more than 2 months duration) eventually died [71,72]. Other important parameters also require further assessment, such as antigen dosages, adjuvants, frequency of applications and inoculation routes, all of which would lead to an optimization of the immunotherapeutic potential of rEmP29 antigen as exploratively documented in the present study. In a similar line of plans, a future evaluation of the immunotherapeutic and immunoprotective potentials of rEmP29 will have to focus on the primary egg infection model that mimics the natural infection in humans more properly.

In human AE, specific IgG antibody levels against Em2(G11) and recEm18 correlate to some extent with the clinical status of the patient [66,73]. In our study, we found that IgG concentrations against Em2(G11) were significantly lower in rEmP29 vaccinated mice in the pre- or post- infection status, when compared to the corresponding control mouse groups immunized or treated with saline solution, saponin alone or with mice that were immunized with rNcMIC1-saponin before challenge.

In our study, administration of adjuvant alone prior to the infection, resulted in a non-specific but still significant (P = 0.002) reduction of the median parasite weight of 38% when compared to mouse groups that received only saline solution. It was reported in a previous study that administration of saponin alone also gave a limited non-specific protection against secondary AE in mice. Similarly, other adjuvants such as CpG oligodeoxynucleotides [30] (CpG ODN), Freund’s complete/Incomplete adjuvants (CFA/IFA) [30], Gerbu [74] or cholera toxin subunit B (CTB) [75] have also been shown to exhibit non-specific protection against larval or adult stages of *E*. *multilocularis*.

In our study, non-specific and significant protection against secondary AE with saponin alone was observed when mice were treated before (pre-infection treatment; exp.2) but not after the infection (post-infection treatment; exp.3). In the same line, it was shown that prophylactic treatment of cotton rat (Sigmodon hispidus) with BCG (Bacillus Calmette-Guerin) entirely and non-specifically inhibited the establishment of *E*. *multilocularis* metacestodes in the intraperitoneal cavity [76,77], however, application of BCG at two weeks post-infection did not limit the development and the proliferation of the parasite[76].

Until now there is no accurate scientific explanation for this phenomenon, however, in addition to the fact that adjuvants generally increase host immunity against infectious diseases, one reason for such a non-specific protection phenomenon may be that saponin or other adjuvants display a direct effect on *E*. *multilocularis* metacestodes or adult worms.

In the present study, we found that the IL-4/IFN-γ mRNA ratios in the spleen of vaccinated mice, or animals treated with rEmP29 saponin formulations, were lower than in spleens of mice treated with saline (infection controls). In addition, corresponding IL-10 and IL-2 expression levels were lower in vaccinated and treated mice compared to saline-treated mice. Thus, higher levels of IFN-γ and reduced expression of IL-10 and IL-2 mRNA might be associated with reduced growth of the parasite in vaccinated and treated mice, and a Th1-oriented response could contribute to the restricted parasite growth of *E*. *multilocularis* within its intermediate host. This is supported by elderly published data where the essential role of cellular immunity in controlling *E*. *multilocularis* infection in humans as well as in mice was confirmed [31,32].

In AE, CD4 (+) CD25 (+) regulatory T cells, have been studied intensively since they are the main IL-10 secreting cells [78,79] and their deficiency abrogates parasite-tolerance. Accumulating evidence from correspondingly selected knockout mice suggested that IL-2 is crucial for the homeostasis and function of CD4 (+) CD25 (+) regulatory T cells *in vivo* [79–85]. This synergic functional relationship between IL-10 and IL-2 may explain the simultaneous down regulation of mRNA levels of both cytokines observed in our study.

The recombinantly expressed rNcMIC1 was used as negative control, since no homologue was found in *E*. *multilocularis* [18]. Recombinant NcMIC1was produced and purified under the same conditions as rEmP29. The antigenic but not the immunosuppressive properties of NcMIC1 were reported [61], but since our results revealed that rNcMIC1 harbors a significant immunomodulatory activity by enhancing IL-10 production; we do not recommend its usage as irrelevant control for future research on *Echinococcus* immunology studies.

To compare the activity level of the immune system between infected mice (independently of the vaccination or immunotherapy status) and non-infected animals, we measured the proliferative activity of spleen cells in response to the mitogen ConA. In line with findings on *E*. *multilocularis* [31,86] and *E*. *granulosus* [87] reported earlier, spleen cells of all infected groups exhibited a significantly decreased responsiveness compared to cells from non-infected mice.

Spleen cells from *E*. *multilocularis*-infected mice treated with saponin at 1 month p.i. exhibited a full responsiveness to ConA-stimulation (99%). Conversely, spleen cells from mice receiving i.p. injection of saponin prior to infection showed a significantly reduced proliferation activity of 32% when compared to non-infected mice. We conclude that the administration of saponin prior to infection had an immuno-stimulating activity by itself, but that this activity was subsequently abolished by the strong immunosuppressive response mediated by the parasite at 3 months p.i.. Upon saponin administration starting at 1 month p.i., the immunosuppression exerted by *E*. *multilocularis* on the host immune system was most likely still moderate, and could not overcome the adjuvant effect. It was previously shown that saponin significantly promoted the ConA, lipopolysaccharide [88] and phytohemagglutinin [89] induced spleen cells proliferation.

In conclusion, we showed that both, vaccination and active immunotherapy employing rEmP29 antigen had a significant inhibitory effect on secondary infection with *E*. *multilocularis* metacestodes in mice, and yielded thus a reduced parasite infection intensity when compared mock-treated control animals. Further studies will aim to assess alternative antigens and also other classes of adjuvants that could improve the immunotherapeutical potential of rEmP29. Furthermore, as vaccination provided partial protection against secondary infection, it will be important now to assess the protection mediated by rEmP29 against primary egg infection.

## Supporting Information

S1 Fig Multiple amino acid sequence alignment of Endophilin B1 (EndB1) proteins. Sequence alignment of *E*. *granulosus* P29 (EgP29), *E*. *granulosus* Endophilin B1 (Eg-EndB1), *E*. *granulosus* Endophilin B1 (Eg-EndB1), *E*. *multilocularis* Endophilin B1 (Em-EndB1), *Homo sapiens* Endophilin B1 isoform 2 (H. s-EndB1-Iso2) and *Homo sapiens* Endophilin B1 isoform 1 (H. s-EndB1-Iso1). The GenBank accession numbers of endophilins are shown in brackets. Strictly conserved residues among the six proteins are highlighted with black boxes. The EgP29 is 238 amino acids in length, and shares 53 (22%) identical amino acid residues with human Endophilin B1 (isoform 1 and 2).
(TIF)
