# Supplementary figures and images for: Prevention and Immunotherapy of Secondary Murine Alveolar Echinococcosis Employing Recombinant EmP29 Antigen
Source: PLoS Negl Trop Dis. 2015 Jun 8;9(6):e0003795. doi: 10.1371/journal.pntd.0003795 (PMC4460070; doi:10.1371/journal.pntd.0003795)

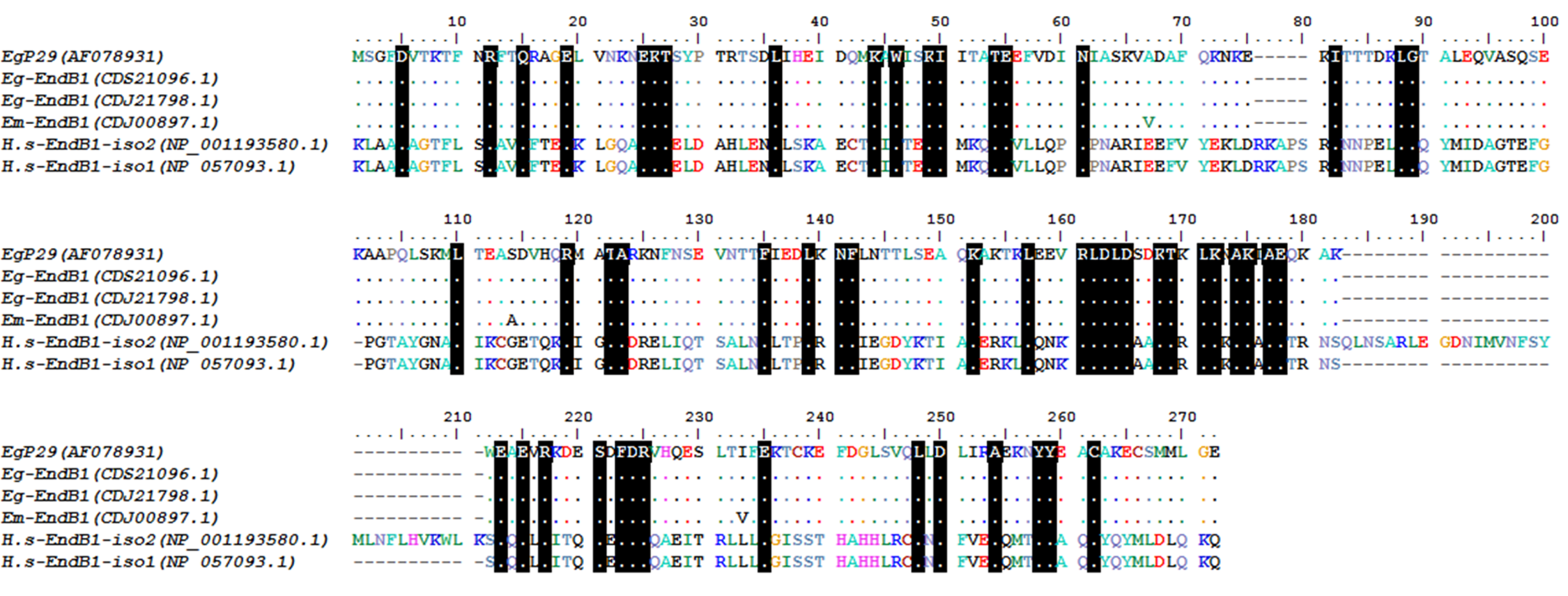

Supplement: S1 Fig — Sequence alignment of E. granulosus P29 (EgP29), E. granulosus Endophilin B1 (Eg-EndB1), E. granulosus Endophilin B1 (Eg-EndB1), E. multilocularis Endophilin B1 (Em-EndB1), Homo sapiens Endophilin B1 isoform 2 (H. s-EndB1-Iso2) and Homo sapiens Endophilin B1 isoform 1 (H. s-EndB1-Iso1). The GenBank accession numbers of endophilins are shown in brackets. Strictly conserved residues among the six proteins are highlighted with black boxes. The EgP29 is 238 amino acids in length, and shares 53 (22%) identical amino acid residues with human Endophilin B1 (isoform 1 and 2). (TIF) [file pntd.0003795.s001.tif]
